# Atopic dermatitis and risk of atrial fibrillation or flutter: A 35-year follow-up study

**DOI:** 10.1016/j.jaad.2019.08.039

**Published:** 2020-12

**Authors:** Sigrun A.J. Schmidt, Morten Olsen, Morten Schmidt, Christian Vestergaard, Sinéad M. Langan, Mette S. Deleuran, Jette L. Riis

**Affiliations:** a Department of Clinical Epidemiology, Aarhus University Hospital, Aarhus, Denmark; b Department of Dermatology, Aarhus University Hospital, Aarhus, Denmark; c Department of Radiology, Aarhus University Hospital, Aarhus, Denmark; d Department of Cardiology, Regional Hospital West Jutland, Herning, Denmark; e Faculty of Epidemiology and Population Health, London School of Hygiene and Tropical Medicine, London, United Kingdom; f Health Data Research UK, London, United Kingdom

**Keywords:** atopic dermatitis, atrial fibrillation, atrial flutter, cohort study, risk factors, validation, CI, confidence interval, DNPR, Danish National Patient Registry, HR, hazard ratio

## Abstract

**Background:**

Atopic dermatitis is characterized by chronic inflammation, which is a risk factor for atrial fibrillation.

**Objective:**

To examine the association between hospital-diagnosed atopic dermatitis and atrial fibrillation.

**Methods:**

Using linked population-based Danish registries, we identified persons with an inpatient or outpatient hospital diagnosis of atopic dermatitis during 1977-2013 and a comparison cohort individually matched to the atopic dermatitis cohort. We followed cohorts until death, emigration, atrial fibrillation diagnosis, or end of study (January 1, 2013). We compared 35-year risk of atrial fibrillation and estimated hazard ratios with 95% confidence intervals using Cox regression, adjusting for birth year and sex. We validated 100 atopic dermatitis diagnoses from a dermatologic department through medical record review.

**Results:**

We included 13,126 persons with atopic dermatitis and 124,211 comparators and followed them for a median of 19.3 years. The 35-year risk of atrial fibrillation was 0.81% and 0.67%, respectively. The positive predictive value of atopic dermatitis diagnoses was 99%. The hazard ratio was 1.2 (95% confidence interval 1.0-1.6) and remained increased after adjusting for various atrial fibrillation risk factors.

**Limitations:**

Analyses were limited to persons with moderate-to-severe atopic dermatitis, and we had no lifestyle data.

**Conclusion:**

Patients with hospital-diagnosed atopic dermatitis have a 20% increased long-term risk of atrial fibrillation, but the absolute risk remains low.

**Capsule Summary**

• We found a 20% increased risk of atrial fibrillation in patients with hospital-diagnosed (moderate-to-severe) atopic dermatitis. This finding might be mediated through persistent systemic inflammation.
• Although the absolute risk is low, the typical early onset of atopic dermatitis provides an opportunity for promoting a heart-healthy lifestyle in these patients.

Atopic dermatitis is a pruritic chronic inflammatory skin disorder.^1^ Prevalence has increased up to 3-fold over the past 3 decades, and the disease now affects 10%-20% of children in industrialized countries,^1^ qualifying atopic dermatitis as the most common chronic childhood disease. However, the common concept that atopic dermatitis is limited to childhood is being abolished. Adult-onset atopic dermatitis is more frequent than previously appreciated, and childhood atopic dermatitis often persists until adulthood or might relapse after long periods with inactive disease.^1^ Thus, up to 10% of adults suffer from atopic dermatitis.^1^

Atrial fibrillation is the most commonly sustained rhythm disorder with a prevalence of 4% in persons aged ≥60 years.^2^ This condition is associated with severe morbidity and mortality (eg, death due to stroke).^2^ Inflammation is a recognized risk factor for atrial fibrillation, as supported by an increased occurrence of atrial fibrillation among patients with rheumatoid arthritis and elevated levels of inflammatory biomarkers.^3^, ^4^, ^5^ The persistent low-grade systemic inflammation associated with atopic dermatitis or the increased prevalence of atrial fibrillation risk factors (eg, obesity, hypertension, and diabetes) among atopic dermatitis patients might thus predispose these patients to atrial fibrillation.^2^, ^6^, ^7^, ^8^, ^9^, ^10^

To provide further evidence on this sparsely examined hypothesis,^9^ we conducted a nationwide population-based 35-year cohort study to examine whether patients with hospital-diagnosed (moderate-to-severe) atopic dermatitis are at long-term increased risk of atrial fibrillation.

## Methods

### Study population

We used the Danish National Patient Registry (DNPR) to identify individuals born in Denmark during January 1, 1947-January 1, 1983, (∼2.7 million people) who received a first-time hospital diagnosis of atopic dermatitis during January 1, 1977-January 1, 2013.^11^ For each hospital discharge or outpatient visit, the physician records 1 primary diagnosis and potentially secondary diagnoses using the International Classification of Diseases, 8th Revision (up through 1993) or 10th Revision (after 1993). We considered all inpatient, outpatient, and emergency room diagnoses of atopic dermatitis using the date of admission or start of outpatient follow-up as the index date. Table I shows definitions for study variables.

**Table I:** Registry codes used to identify study variables

Variable	Codes
Atopic dermatitis	ICD-8: 691; ICD-10: L20
Azathioprine	ATC code: L04AX01; procedure code: BWHB83
Methotrexate	ATC code: L01BA01, L04AX03; procedure code: BWHA115
Cyclosporine	ATC code: L04AD01; procedure code: BOHJ20
Mycophenolate	ATC code: L04AA06; procedure code: BOHJ22
Phototherapy	Procedure code: BNGA1, BNGA2, BNGA3, BNGA4
Atrial fibrillation and flutter	ICD-8: 42793, 42794; ICD-10: I48
Allergic asthma	ICD-8: 493; ICD-10: J450
Allergic rhinitis	ICD-8: 50709; ICD-10: J301-J304
Chronic obstructive pulmonary disease or nonallergic asthma	ICD-8: 491, 492; ICD-10: J41, J42, J43, J44, J45 (except J450), J46; ATC code: R03 (at least 2 prescriptions) except if the person has a prevalent allergic asthma diagnosis
Cardiovascular disease, including structural valve problems, hypertension, ischemic heart disease, and heart failure	ICD-8: 393-398, 400-404, 410-414, 425, 42709, 42710, 42711, 42719, 78249; ICD-10: I05-I09, I10-I15, I20-I25, I34-I37, I390, I393, I42 (excluding I426 included below), I43, I50, I511A, Q22; ATC code: C01DA, C02, C03,C07, C08, C09, B01AC04, B01AC06, N02BA01; Procedure code: 30009, 30019, 30029, 30039, 30049, 30059, 30069, 30079, 30089, 30099, 30109, 30119, 30120, 30129, 30139, 30149, 30159, 30169, 30179, 30189, 30199, 30200 30300, 30310, 30320, 30330, 30340, 30350, 30360, 30600, 30620, 30640, 30660, 30700, 30701, 30709, 30719, 30720, 30729, 30740, 30780, 30799, 30800, 30810, 30910, 30920, 30925, 30939, 30959, 30990, 31100, 31101, 31119, 31129, 31130, 31180, 31199, 31200, 31210, 31220, 31229, 31230, 31249, 31259, 31268, 31269, 31280, 31299, 31310, 30350, 30354, 30240, KFNA-E, KFNH20, KFM (excluding KFMA32, KFMD10-14, KFMH10), KFK (excluding KFKA32, KFKC70, KFKH10), KFG (excluding KFGA32) KFJE (excluding KFJE42), KFJF, KFJW, KFNG, KFNF
Rheumatic disease	ICD-8: 28709, 69609, 712, 716, 734, 446; ICD-10: D690B, G737, G058A, H221B, I328A, I328B, I398C, I398E, I418A, I528A, I776, L931, L932, L95, M05-M07, M30-M36, M45, M793, N085, N164
Sleep apnea	ICD-10: G473
Obesity	ICD-8: 277; ICD-10: E65-E66
Hyperthyroidism	ICD-8: 242; ICD-10: E05, H062, E060, E062; ATC code: H03B
Chronic kidney disease	ICD-8: 584, 792, 9977, Y9509; ICD-10: L298C, G638A, E853B, T825A, T825B, T825C, T856C, I120, I131, I132, I770, N165, N180, N183, N184, N185, N188, N189, N19, T824, T861, Z49, Z94, Z992, T817E1; procedure code: KJAK10, KJAK11, KJAK13, KJAK14, KTJA30, KTJA32, KTJA35, KKAS, BJFD2, BJFZ, BJKB, BUFC1, BWDC5, ZZ0151A, ZZ4341, ZZ4342, ZZ4343, ZZ4346, ZZ4347, ZZ4348, ZZ4350, 57480, 57490, 87409, 87419, 87420, 87430, 87431, 87432, 87440, 92390, 92400, 94300, 94340
Diabetes mellitus	ICD-8: 249-250; ICD-10: E10-E14, H360, O24 (except O244), H360, N083, DG632; ATC code: A10
Alcoholism-related disorder or prescription	ICD-8: 291, 303, 57109-57110, 57710, 979, 980; ICD-10: F10, G312, G621, G721, I426, K292, K700, K703, K860, R780, T510, T519, Z721; ATC code: N07BB01

Only primary and secondary diagnoses from the Danish National Patient Registry are included.

*ATC*, Anatomical therapeutic chemical; *ICD8*, International Classification of Diseases, 8th Revision; *ICD10*: International Classification of Diseases, 10th Revision.

We used the Civil Registration System^12^ to sample a comparison cohort that included 10 individuals from the general population matched to each atopic dermatitis patient by sex and birth year. We assigned persons in the comparison cohort with the same index date as their corresponding atopic dermatitis patient.

### Validation

We examined the validity of 100 randomly selected inpatient and outpatient diagnoses of atopic dermatitis from the Department of Dermatology, Aarhus University Hospital, that occurred during 1977-2016. One author (Dr Riis) scrutinized patients' medical records using as reference standard the diagnosis stated by the treating physician in the medical record.

### Atrial fibrillation or flutter

We used the DNPR to obtain information on all inpatient or outpatient primary or secondary diagnoses of atrial fibrillation in the study population. Because of overlapping pathophysiology, we included both atrial fibrillation and flutter.^13^, ^14^ To ensure that only incident diagnoses were considered, we excluded persons in the study cohorts who had atrial fibrillation recorded before the index date.

### Patient characteristics

We considered patients to have severe atopic dermatitis if they filled a prescription for azathioprine, cyclosporine, mycophenolate, or methotrexate, which are used in systemic atopic dermatitis treatment,^1^ or if they were admitted with atopic dermatitis coded as the primary reason for admission. We identified systemic treatments through the Danish National Prescription Registry, which was established in 1995 and includes records of all prescription drugs dispensed at Danish pharmacies, and classified them according to the anatomical therapeutic chemical classification.^15^ Because we were limited to patients in hospital-based settings, we considered all patients to have at least atopic dermatitis of moderate severity at the outset. We included severity as a time-updated variable, ie, patients contributed person-time in the moderate category switching to the severe category for the remainder of the follow-up if and when they fulfilled the definition for severe atopic dermatitis. As an alternative measure of severity and activity, we used number of atopic dermatitis contacts (1, 2-4, 5-7, ≥8). In this analysis, the index date was the first, second, fifth, and eighth contact, respectively, for atopic dermatitis patients and their matched comparators. We also included diagnoses of allergic asthma or rhinitis as a measure of atopic multimorbidity.

We used the DNPR to identify the following potential atrial fibrillation risk factors^2^, ^4^, ^8^: chronic obstructive pulmonary disease, cardiovascular disease (ischemic heart disease, heart failure, hypertension, and structural valve problems), rheumatic disease, sleep apnea, hospital-diagnosed obesity, hyperthyroidism, chronic kidney disease, diabetes, and alcohol-related disease. We used the DNPR^11^ and Danish National Prescription Registry^15^ to identify procedures and treatments as disease proxies to increase completeness when relevant (eg, antidiabetic drugs as a proxy for diabetes). We included these conditions because they might be more prevalent among atopic dermatitis patients as a result of immune dysregulation, shared pathophysiology, adverse effects of treatment, or affected lifestyle choices,^1^, ^6^, ^7^, ^9^ thereby explaining an association with atrial fibrillation. In the main analysis, we considered these covariables as potential confounders, including records available before the index date. In additional analyses, we considered the possibility that these covariables were mediators by time-updating these variables with information recorded after the start of follow-up. Thus, a person was considered to have a given disease from the first registry record defining that disease and onwards.

We used education registries from Statistics Denmark^16^ to identify the highest educational level on the index date, classified as short-term (7-10 years), medium-term (11-12 years), or long-term (≥13 year) education.

### Statistical analysis

For the validation sample, we computed the positive predictive value with 95% confidence intervals (CIs; on the basis of the Wilson score method^17^) as the percentage with confirmed diagnoses.

We followed cohorts from the index date until atrial fibrillation diagnosis, emigration, death, or the end of the study (January 1, 2013), whichever occurred first. We produced descriptive statistics for the cohorts. We plotted the cumulative incidence of atrial fibrillation for atopic dermatitis and comparison cohorts, with death as a competing risk. We used Cox proportional hazards regression stratified on the matched set to compute hazard ratios (HRs) with 95% CIs as a measure of the relative risk of the association between atopic dermatitis and atrial fibrillation or flutter. We used time from the index date as the underlying time scale. To explore the role of certain variables as mediators, we fitted several regression models of increasing complexity. Model 1 was unadjusted, accounting only for matched factors. Model 2 adjusted additionally for baseline atrial fibrillation risk factors, and model 3 adjusted also for educational level for those with nonmissing information for this variable (ie, a complete case analysis).

In stratified analyses, we examined whether the association varied by sex and presence of allergic asthma or rhinitis. We also examined results for subgroups defined by age at first atopic dermatitis contact (0-19, 20-39, ≥40 years), severity, and number of atopic dermatitis hospital contacts. We performed severity analyses with delayed entry until January 1, 1996, to ensure at least 1 year of prescription history.^15^

We performed 3 sensitivity analyses. We repeated model 2 with atrial fibrillation risk factors included as time-updated covariates (mediation analysis). We repeated the main analyses with delayed entry until January 1, 1996, in all comparisons. We repeated severity analyses adding phototherapy as another criterion for severe atopic dermatitis.

We verified the assumption of proportional hazards by visual inspection of log (–log [survival]) versus log (survival time) plots. Analyses were performed with Stata 14.2 (StataCorp LP, Texas, US). The study was approved by the Danish Data Protection Agency (record no. 2013-41-2237; 2016-051-000001). Danish legislation does not require approval by an ethical review board or informed consent from patients for registry-based studies. The Danish Patient Safety Authority approved access to medical records for the validation of diagnoses (record no. 3-3013-1526/1/).

## Results

We were able to retrieve all, but 1, medical records for the validation sample. Medical review confirmed 98 of 99 diagnoses, yielding a positive predictive value of 99% (95% CI 95%-100%).

We identified 13,144 eligible persons with atopic dermatitis and 124,487 matched comparators and subsequently excluded 18 atopic dermatitis patients (and 165 comparators plus an additional 111 comparators with prevalent atrial fibrillation). Distribution of characteristics was quite similar among atopic dermatitis patients and comparators (Table II). Male persons accounted for 43% of both populations. Median age was 19 (interquartile range 6-29) years. Allergic asthma or rhinitis and chronic obstructive pulmonary disease were more common in atopic dermatitis patients.

**Table II:** Characteristics at cohort entry for persons with hospital-diagnosed atopic dermatitis compared with a matched comparison cohort, Denmark, 1977-2013

Characteristic	Atopic dermatitis, n (%)	Matched comparators, n (%)
Total	13,126 (100)	124,211 (100)
Sex		
Male	5630 (43)	54,024 (43)
Female	7496 (57)	70,187 (57)
Birth year		
1947-1956	1266 (10)	11,800 (9)
1957-1966	2631 (20)	24,525 (20)
1967-1982	9229 (70)	87,886 (71)
Age at start of follow-up, y		
0-19	6509 (50)	64,274 (52)
20-39	5208 (40)	47,165 (38)
40-63	1409 (11)	12,772 (10)
Allergic asthma or rhinitis	842 (6)	1002 (1)
Sleep apnea	6 (0)	54 (0)
Hospital-diagnosed obesity	88 (1)	835 (1)
Rheumatic disease	110 (1)	498 (0)
Chronic kidney disease	35 (0)	87 (0)
COPD	1536 (12)	5648 (5)
Cardiovascular disease	459 (3)	3149 (3)
Diabetes mellitus	84 (1)	771 (1)
Hyperthyroidism	32 (0)	325 (0)
Alcohol-related disease	243 (2)	1681 (1)
Educational level		
Short-term education	2608 (20)	23,380 (19)
Medium-term education	5172 (39)	53,272 (43)
Long-term education	5109 (39)	45,512 (37)
Missing	237 (2)	2047 (2)

All variables in the table are measured at start of follow-up.

*COPD*, Chronic obstructive pulmonary disease.

**Fig 1:**
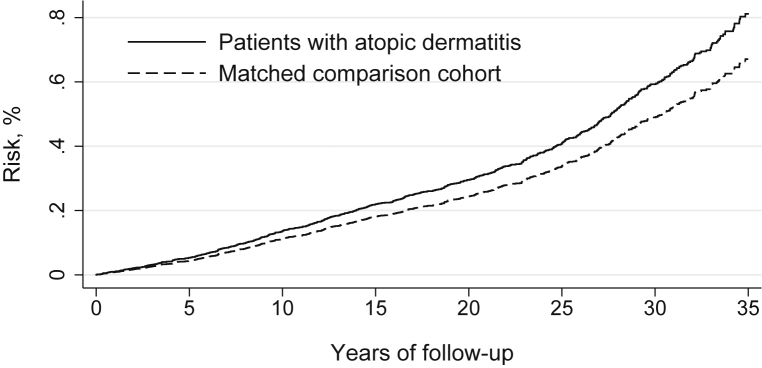
Risk of atrial fibrillation or flutter in patients with hospital-diagnosed atopic dermatitis and a matched comparison cohort, adjusted for birth year and sex, Denmark, 1977-2013.

Median follow-up was 19.3 years (total 2,787,675 person-years). The cumulative incidence of atrial fibrillation after 35 years of follow-up was 0.81% for the atopic dermatitis cohort and 0.67% for the comparison cohort (Fig 1). The corresponding unadjusted HR was 1.2 (95% CI 1.0-1.6) for atopic dermatitis patients versus the matched comparison cohort (Table III). Increasing level of adjustment had no substantial effect on estimates (HR 1.2, 95% CI 0.9-1.5).

**Table III:** Observations, events, person-years, rates, and HRs of atrial fibrillation or flutter for persons with hospital-diagnosed atopic dermatitis compared with a matched comparison cohort, Denmark, 1977-2013

Category	Observations, n	Events, n	Person-years	Rate, per 100,000 population	Model 1, HR (95% CI)∗	Model 2, HR (95% CI)†	Model 3, HR (95% CI)‡
Overall							
Comparators	124,211	631	2,530,240	24.9	1 (Reference)	1 (Reference)	1 (Reference)
Atopic dermatitis	13,126	80	257,435	31.1	1.2 (1.0-1.6)	1.2 (0.9-1.5)	1.2 (0.9-1.5)
Sex							
Male							
Comparators	54,024	402	1,188,398	33.8	1 (Reference)	1 (Reference)	1 (Reference)
Atopic dermatitis	5630	43	119,291	36.0	1.0 (0.7-1.4)	1.0 (0.7-1.3)	1.0 (0.7-1.4)
Female							
Comparators	70,187	229	1,341,842	17.1	1 (Reference)	1 (Reference)	1 (Reference)
Atopic dermatitis	7496	37	138,143	26.8	1.6 (1.1-2.3)	1.6 (1.1-2.3)	1.6 (1.1-2.3)
Age at start of follow-up							
0-19 years							
Comparators	64,274	190	1,716,596	11.1	1 (Reference)	1 (Reference)	1 (Reference)
Atopic dermatitis	6509	29	170,203	17.0	1.6 (1.1-2.4)	1.6 (1.1-2.4)	1.6 (1.1-2.4)
20-39 years							
Comparators	47,165	253	698,660	36.2	1 (Reference)	1 (Reference)	1 (Reference)
Atopic dermatitis	5208	28	74,928	37.4	1.0 (0.7-1.6)	1.0 (0.7-1.5)	1.0 (0.7-1.5)
40-63 years							
Comparators	12,772	188	114,984	163.5	1 (Reference)	1 (Reference)	1 (Reference)
Atopic dermatitis	1409	23	12,304	186.9	1.1 (0.7-1.8)	1.1 (0.7-1.7)	1.1 (0.7-1.7)
Severity							
Moderate							
Comparators	76,169	300	911,576	32.9	1 (Reference)	1 (Reference)	1 (Reference)
Atopic dermatitis	8357	38	98,463	38.6	1.2 (0.8-1.7)	1.1 (0.8-1.6)	1.1 (0.8-1.6)
Severe							
Comparators	45,873	280	637,486	43.9	1 (Reference)	1 (Reference)	1 (Reference)
Atopic dermatitis	5023	39	69,348	56.2	1.3 (0.9-1.8)	1.2 (0.9-1.8)	1.3 (0.9-1.8)
Allergic asthma or rhinitis							
No							
Comparators	94,260	435	1,669,224	26.1	1 (Reference)	1 (Reference)	1 (Reference)
Atopic dermatitis	9979	47	170,487	27.6	1.0 (0.8-1.4)	1.0 (0.7-1.4)	1.0 (0.7-1.4)
Yes							
Comparators	41,472	196	861,016	22.8	1 (Reference)	1 (Reference)	1 (Reference)
Atopic dermatitis	4347	33	86,948	38.0	1.7 (1.2-2.5)	1.6 (1.1-2.4)	1.7 (1.1-2.4)
No. atopic dermatitis contacts							
1							
Comparators	124,211	417	1,787,221	23.3	1 (Reference)	1 (Reference)	1 (Reference)
Atopic dermatitis	13,126	45	182,210	24.7	1.0 (0.8-1.4)	1.0 (0.8-1.4)	1.0 (0.8-1.4)
2-4							
Comparators	40,452	162	619,928	26.1	1 (Reference)	1 (Reference)	1 (Reference)
Atopic dermatitis	4277	27	62,925	42.9	1.6 (1.1-2.5)	1.6 (1.0-2.4)	1.6 (1.0-2.4)
5-7							
Comparators	7653	37	79,909	46.3	1 (Reference)	1 (Reference)	1 (Reference)
Atopic dermatitis	806	3	7964	37.7	0.7 (0.2-2.4)	0.8 (0.3-2.8)	0.9 (0.3-2.8)
≥8							
Comparators	3052	15	43,182	34.7	1 (Reference)	1 (Reference)	1 (Reference)
Atopic dermatitis	327	5	4336	115.3	3.2 (1.1-8.8)	3.9 (1.3-12.0)	4.0 (1.3-12.6)

*CI*, Confidence interval; *HR*, hazard ratio.

∗ Unadjusted, stratified by matched set to account for matching factors (birth year, sex, index date).

† Additionally adjusted for atrial fibrillation risk factors at baseline: chronic obstructive pulmonary disease, cardiovascular disease, rheumatic disease, sleep apnea, hospital-diagnosed obesity, hyperthyroidism, chronic kidney disease, diabetes mellitus, and alcohol-related disease.

‡ Additionally adjusted for educational level (complete-case analysis).

We found some evidence of variation by sex (HR 1.6 for female vs 1.0 for male patients), diagnosis age (HR 1.6 for 0-19 years vs 1.0-1.1 for older age groups), coexisting diagnosis of allergic asthma or rhinitis (HR 1.7 for presence vs 1.0 for absence of diagnosis), and number of contacts (HR 4.0 for ≥8 contacts vs 1.0 for <2 contacts) for an atopic dermatitis diagnosis (Table III). The HR was 1.1 (95% CI 0.8-1.6) for moderate and 1.3 (95% CI 0.9-1.8) for severe atopic dermatitis. These potential differences were also apparent on the absolute scale comparing unadjusted rates (Table III).

Estimates were attenuated but remained increased in the mediation analysis incorporating time-varying information for covariables. (Table IV). There were no substantial changes in estimates when analyzing with delayed entry or when using phototherapy as a proxy for severe atopic dermatitis in addition to other systematic therapies and admissions (Table IV).

**Table IV:** Sensitivity analyses of atrial fibrillation or flutter for persons with hospital-diagnosed atopic dermatitis compared with a matched comparison cohort, Denmark, 1977-2013

Characteristic	Sensitivity analysis 1∗	Sensitivity analysis 2†			Sensitivity analysis 3‡		
	Model 2	Model 1	Model 2	Model 3	Model 1	Model 2	Model 3
Overall	1.0 (0.8-1.3)	1.2 (1.0-1.6)	1.2 (0.9-1.5)	1.2 (0.9-1.5)	NA	NA	NA
Sex							
Male	0.9 (0.6-1.2)	1.0 (0.7-1.4)	1.0 (0.7-1.3)	1.0 (0.7-1.3)	NA	NA	NA
Female	1.5 (1.0-2.1)	1.6 (1.1-2.3)	1.6 (1.1-2.3)	1.6 (1.1-2.3)	NA	NA	NA
Diagnosis age, y							
0-19	1.5 (1.0-2.4)	1.5 (1.0-2.3)	1.5 (1.0-2.3)	1.5 (1.0-2.3)	NA	NA	NA
20-39	0.9 (0.6-1.3)	1.1 (0.7-1.6)	1.0 (0.7-1.5)	1.0 (0.7-1.6)	NA	NA	NA
40-63	0.9 (0.6-1.5)	1.1 (0.7-1.8)	1.1 (0.7-1.7)	1.1 (0.7-1.7)	NA	NA	NA
Severity							
Moderate	1.1 (0.7-1.5)	1.2 (0.8-1.7)	1.1 (0.8-1.6)	1.1 (0.8-1.6)	1.2 (0.9-1.7)	1.2 (0.8-1.7)	1.2 (0.8-1.7)
Severe	1.1 (0.7-1.5)	1.3 (0.9-1.8)	1.2 (0.9-1.8)	1.3 (0.9-1.8)	1.2 (0.9-1.7)	1.2 (0.9-1.7)	1.2 (0.9-1.7)
Allergic asthma or rhinitis							
No	0.9 (0.7-1.2)	1.0 (0.8-1.4)	1.0 (0.7-1.4)	1.0 (0.7-1.4)	NA	NA	NA
Yes	1.4 (0.9-2.2)	1.7 (1.1-2.4)	1.6 (1.1-2.4)	1.6 (1.1-2.4)	NA	NA	NA
No. atopic dermatitis of contacts							
<2	0.9 (0.7-1.3)	1.1 (0.8-1.5)	1.0 (0.8-1.4)	1.1 (0.8-1.4)	NA	NA	NA
2-4	1.3 (0.8-2.0)	1.6 (1.1-2.5)	1.5 (1.0-2.4)	1.5 (1.0-2.4)	NA	NA	NA
5-7	1.1 (0.3-4.4)	0.5 (0.1-2.1)	0.6 (0.1-2.4)	0.6 (0.1-2.5)	NA	NA	NA
≥8	2.9 (0.6-14.1)	3.2 (1.1-8.8)	3.9 (1.3-12.0)	4.0 (1.3-12.6)	NA	NA	NA

Values are HR (95% CI). Models were based on a Cox regression model stratified by matched set to account for matching factors (birth year, sex, index date). Model 1 was unadjusted. Model 2 additionally adjusted for atrial fibrillation risk factors chronic obstructive pulmonary disease, cardiovascular disease, rheumatic disease, sleep apnea, hospital-diagnosed obesity, hyperthyroidism, chronic kidney disease, diabetes mellitus, and alcohol-related disease. Model 3 as additionally adjusted for educational level (complete-case analysis).

*CI*, Confidence interval; *HR*, hazard ratio; *NA*, not applicable.

∗ Repeated model 2 with atrial fibrillation risk factors included as time-updated covariates (mediation analysis).

† Repeated the main analyses with delayed entry until January 1, 1996, in all comparisons.

‡ Repeated severity analyses adding phototherapy as another criterion for severe atopic dermatitis.

## Discussion

This long-term follow-up study shows evidence that hospital-diagnosed atopic dermatitis is associated with a 20% increased relative risk of atrial fibrillation. Characteristics associated with particular high-risk estimates were female sex, young age at first hospital diagnosis, and indicators for severe disease (eg, atopic multimorbidity, frequent hospital contact).

To the best of our knowledge, this topic has only been examined in 1 previous epidemiologic study.^9^ In a population-based UK cohort study, adult persons with versus without atopic dermatitis had an HR for atrial fibrillation of 1.11 (99% CI 1.04-1.18), increasing to 1.17 (99% CI 1.08-1.27) for moderate and 1.38 (99% CI 1.17-1.62) for severe disease.^9^ There was no evidence that age, sex, or asthma modified the association. Although this study was limited to adults and had a median follow-up of only 5.1 years, our results are in accordance with these findings for moderate-to-severe disease. Likewise, adjustment for potential mediators of the association explained findings only partly.

A potential mechanism underlying the observed association is systemic inflammation, similar to that presumed to link psoriasis and rheumatic disorders to atrial fibrillation.^3^, ^4^, ^5^, ^18^ Arrhythmogenic effects of atopic dermatitis treatments is also possible, although evidence for such adverse effects is limited.^19^, ^20^, ^21^, ^22^ The more pronounced association for those with many hospital contacts and coexisting atopic conditions supports these mechanisms. Study size precluded analyses of the effect of individual systemic drugs on atopic dermatitis. Psychosocial and unhealthy lifestyle factors (eg, stress, elevated blood pressure, smoking, diabetes, and hypercholesterolemia) could play a role as well,^6^, ^7^ although obesity is not more prevalent in European patients with atopic dermatitis.^10^ The slight attenuation from adjustment for various atrial fibrillation risk factors could support this hypothesis.

The population-based design in a universal health care system with virtually complete follow-up of patients eliminates selection bias in our study.^12^ Furthermore, follow-up was longer than the UK study (median 19.3 vs 5.1 years).^9^ Nevertheless, the highest-possible attained age was 30-65 years, which is relatively low, considering the usual age of onset of atrial fibrillation.^23^ Furthermore, some patients might have had atopic dermatitis before entering our study. Because of such onset misclassification, atrial fibrillation risk factors recorded at baseline might be intermediate steps (rather than confounders) linking atopic dermatitis to atrial fibrillation.

The validity of atopic dermatitis diagnoses was found to be high in our validation sample at a single dermatology department. Although the positive predictive value might be unrepresentative of other departments; misclassification of atopic dermatitis in the entire study population is unlikely to depend on the outcome (atrial fibrillation) because data were prospectively collected. Such nondifferential misclassification tends to produce underestimates and can therefore not explain an observed association. Furthermore, although some patients might actually have had other cutaneous conditions associated with atrial fibrillation (eg, venous insufficiency or pruritus in chronic kidney disease), these conditions are rare in young persons (50% were <20 years of age at index diagnosis) and had the HR of atrial fibrillation associated with atopic dermatitis.

Limited variation by severity could result from misclassification, as we lacked clinical information on severity or activity. Furthermore, we defined severe disease by systemic treatments, which could oppose the proposed mechanism by decreasing inflammation and thus lead to underestimates for the severe category in our study. Finally, because most patients with atopic dermatitis receive diagnoses outside the hospital setting, our study already represents the most severe end of the disease spectrum. This incompleteness might have affected the possibility to detect variation by severity and potential generalizability to mild atopic dermatitis.

The positive predictive value of atrial fibrillation diagnoses in the DNPR is high (92%-99%).^24^, ^25^, ^26^, ^27^ We did not distinguish between atrial fibrillation patterns (paroxysmal, persistent, or permanent) or between atrial fibrillation and flutter. Because atrial fibrillation accounts for >90% of patients registered with these codes,^26^ our results are likely driven by this arrhythmia. Still, as atrial fibrillation and flutter share risk factors and to some degree pathophysiology,^13^, ^14^ we expect the results to apply to both. Regarding completeness, most patients with atrial fibrillation receive their diagnoses during a hospital admission or at a hospital outpatient clinic according to Danish guidelines,^28^ and few cardiologists work outside the public hospital system in Denmark. However, because of regular follow-up of patients with severe atopic dermatitis, opportunity for an atrial fibrillation diagnosis might be greater for matched comparators (ie, ascertainment bias).

We adjusted for education and several comorbidities but cannot exclude misclassification of these mediators, residual confounding and confounding from unmeasured variables.

Last, statistical uncertainty (as measured by the width of CIs) should be considered.^29^ Although our data are best compatible with a 20% increase in the rate of atrial fibrillation among atopic dermatitis patients, our data are reasonably compatible with a small (10%) decrease to a substantially elevated (50%) increase in relative risk. Of note, subgroup analyses should be interpreted cautiously because the lower number of events reduced the statistical precision.

In conclusion, patients with hospital-diagnosed (moderate-to-severe) atopic dermatitis have a 20% increased long-term risk of atrial fibrillation compared with the general population. Although the clinical implications are limited by a low absolute risk of atrial fibrillation, the typical early onset of atopic dermatitis could provide clinicians with a unique opportunity for promoting a heart-healthy lifestyle to reduce risk for cardiovascular disease, including atrial fibrillation.
